# Editorial: Update in endoscopic and transcranial approaches for skull base meningiomas

**DOI:** 10.3389/fneur.2024.1484978

**Published:** 2024-10-21

**Authors:** Edgar G. Ordóñez-Rubiano, Sabino Luzzi, Joao P. Almeida, William Omar Contreras López, Emanuele La Corte, Matías Baldoncini, Alvaro Campero

**Affiliations:** ^1^Department of Neurosurgery, Fundación Universitaria de Ciencias de la Salud (FUCS), Hospital de San José—Sociedad de Cirugía de Bogotá, Bogotá, Colombia; ^2^Department of Neurosurgery, Hospital Universitario Fundación Santa Fe de Bogotá, Bogotá, Colombia; ^3^Department of Clinical-Surgical, Diagnostic and Pediatric Sciences, University of Pavia, Pavia, Italy; ^4^Neurosurgery Unit, Department of Surgical Sciences, Fondazione IRCCS Policlinico San Matteo, Pavia, Italy; ^5^Department of Neurosurgery, Mayo Clinic, Jacksonville, FL, United States; ^6^Functional Neurosurgery, NEMOD International Neuromodulation Center, Clínica Foscal Internacional, UNAB University, Bucaramanga, Colombia; ^7^Neurosurgery and Neurotraumatology Unit, Department of Neurosciences, IRCCS Ospedale Policlinico San Martino, Genova, Italy; ^8^School of Medicine, Laboratory of Microsurgical Neuroanatomy, Second Chair of Gross Anatomy, University of Buenos Aires, Buenos Aires, Argentina; ^9^Department of Neurological Surgery, Hospital San Fernando, Buenos Aires, Argentina; ^10^Department of Neurological Surgery, Padilla Hospital, San Miguel de Tucumán, Argentina

**Keywords:** meningioma, skull base, neurosurgery, surgery, computer-assisted

## Introduction

This editorial frames the aims and objectives of our Research Topic, while also placing the findings in a broader context. There were eight published articles in this Research Topic, including two review articles, four original research studies, and two case reports. Here we discuss the contributing articles of the Research Topic, providing insight into how they collectively advance understanding in the development of endoscopic and microscopic techniques for resection of skull base meningiomas.

## Research Topic discussion

### Sellar and parasellar region

**Figure 1:**
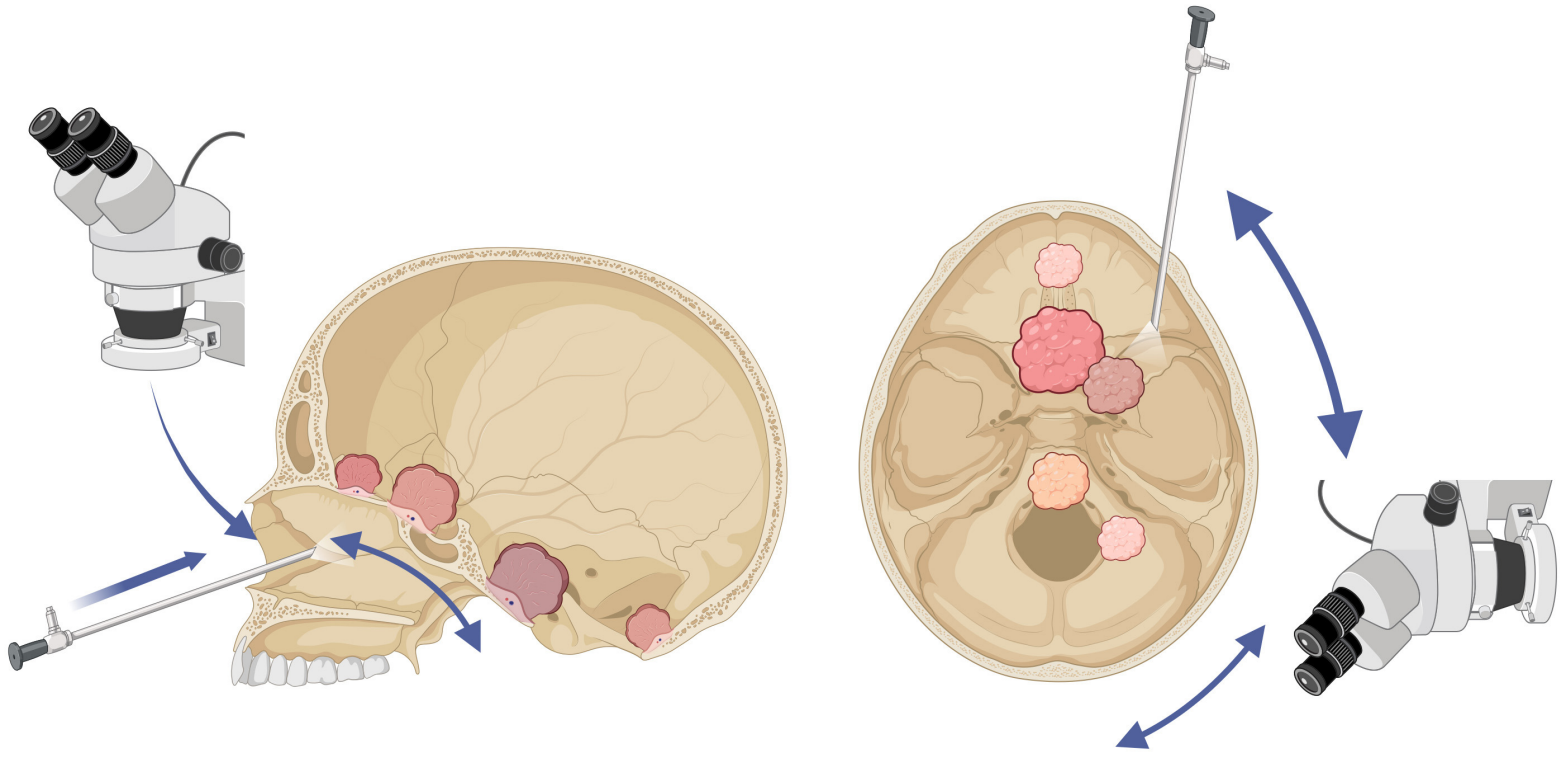
Illustration of surgical approaches to skull base meningiomas. On the **(left)**, there is a representation of the use of the microscope and the endoscope for endonasal approaches to the anterior, middle, and posterior fossa. On the **(right)**, there is an illustration of the transorbital endoscopic approach to the middle and posterior fossa, as well as the utility of both the endoscope and the microscope for lateral approaches to the middle and posterior fossa. Edgar G. Ordóñez-Rubiano^©^, created with: www.biorender.com.

For macroadenomas, the most suitable approaches remain transsphenoidal microscopic and endonasal endoscopic techniques. Kong et al. showed that the rates of tumor removal and postoperative hormone remission were comparable with both techniques. However, they showed that the microscopic group experienced shorter surgery times, reduced blood loss during the procedure, quicker recovery of the sense of smell, and lower average surgery costs (Kong et al.). On the other hand, for lesions like planum sphenoidale and tuberculum sellae meningiomas, the approach remains controversial. Although the traditional transcranial approach remains the gold standard for tuberculum sellae meningiomas, recent decades have seen a growing body of literature supporting endonasal resections for specific scenarios (Figure 1). These include cases involving midline tumors smaller than 3.5 cm, with no invasion of the optic canal and no encasement of blood vessels (1). Feng et al. showed that both extended endonasal endoscopic approach (EEEA) and craniotomy can effectively remove tuberculum sellae meningiomas, with similar recurrence or progression rates and progression-free survival in both groups. Despite no significant differences in the extent of resection and visual outcomes, the EEEA group showed a tendency toward better outcomes. CSF leakage was more frequent in the EEEA group, while cranial nerve injury rates were significantly higher in the craniotomy group (Feng et al.). However, the incidence of olfactory dysfunction is high in patients following endoscopic transsphenoidal resection of the sellar region.

Zhou et al. performed a cross-sectional study of 158 patients where they found a higher incidence of olfactory dysfunction in patients after an endoscopic endonasal approach. The main influencing factors were the formation of blood scabs, nasal adhesions, cerebrospinal fluid rhinorrhea, and the duration of the operation (Zhou et al.). For this, they recommend starting saline nasal irrigation 24 h after the removal of bilateral nasal packing. This practice helps clean the nasal cavity, improve the function of nasal mucosal cilia, and promote the repair of the nasal mucosa and wound healing (Zhou et al.).

### Posterior fossa

As reviewed by Baldoncini et al., many corridors have been described not only for foramen magnum meningiomas (FMM) but for other meningiomas located in the posterior fossa. Awyono et al. described an endoscopic endonasal trans-posterior clinoid approach that avoids all the disadvantages of the traditional transcranial approach, offering an early method to devascularize and detach the tumor from its attachment at the posterior clinoid process. They reported this approach to be safe and effective, making it a viable alternative for resecting meningiomas in this location (Awyono et al.). For FMM, Baldoncini et al. conclude that gross-total resection of meningiomas in this region remains the gold standard of treatment and should be pursued whenever possible. In selected cases of anterior midline foramen magnum meningiomas, the endoscopic transclivus approach can be useful. However, it should be performed in centers with advanced expertise in endoscopic skull base surgery, considering the increased risk of postoperative cerebrospinal fluid leaks (Baldoncini et al.).

On the other hand, the advent of anterior and lateral microscopic and endoscopic approaches has opened the armamentarium of neurosurgeons to approach the posterior fossa. The recent advancement of the transorbital corridor has been demonstrated to be a feasible way to approach the petrous apex and the posterior fossa (2). Kwon et al. performed a cadaveric study comparing the surgical maneuverability and brainstem exposure of the transcranial vs. the endoscopic transorbital petrosectomy. The authors concluded that the endoscopic transorbital approach could be a valid surgical option in selected cases, providing a direct ventral route to the brainstem. Unfortunately, they showed that it offers less surgical maneuverability, a wider angle of attack, and a longer surgical depth compared to other traditional approaches (Kwon et al.).

Finally, Li et al. described a multi-corridor hybrid surgery that allows for one-stage total tumor removal of a dumbbell-shaped tumor extending from the middle fossa to the posterior fossa. This hybrid technique combines multiple surgical corridors to achieve comprehensive resection of the tumor, which can be especially effective for complex skull-base lesions. The technique shows promise in improving patient outcomes by offering a more efficient and less invasive surgical option (Li et al.).

### Future perspectives

Gómez Amarillo et al. have nicely reviewed the use of augmented reality (AR) for resection of intracranial meningioma. AR has demonstrated the potential to enhance neurosurgeons' awareness of critical neurovascular structures, aiding in tumor dissection, and improving overall surgical outcomes. They remark that the existing literature on intraoperative AR is still limited, but ongoing research suggests that AR can be a valuable tool for guiding surgeons on a case-by-case basis. As advancements in artificial intelligence progress, integrating AR and mixed reality into neurosurgical procedures is expected to enhance the safety and effectiveness of meningioma resections, addressing the ongoing need to improve surgical results (Gómez Amarillo et al.).

## Conclusion

The primary advancements in endoscopic and transcranial approaches are focused on improving minimally invasive techniques, sometimes applying hybrid techniques. There are no perfect approaches for a selected skull base meningioma; however, there is an increasing number of options to select. The development of new technologies like AR will guide neurosurgeons to improve these approaches.
